# Predictive values of biochemical markers as early indicators for severe COVID-19 cases in admission

**DOI:** 10.2217/fvl-2020-0319

**Published:** 2021-05-11

**Authors:** Emin Gemcioglu, Mehmet Davutoglu, Ramis Catalbas, Berkan Karabuga, Enes Kaptan, Adalet Aypak, Ayse K Kalem, Mustafa Özdemir, Necati Y Yeşilova, Emra A Kalkan, Musa Civak, Orhan Kücüksahin, Abdulsamet Erden, Ihsan Ates

**Affiliations:** 1 ^1^Ankara City Hospital, Department of Internal Medicine, Ankara 06800, Turkey; 2 ^2^Yıldırım Beyazıt University School of Medicine, Department of Internal Medicine, Ankara 06800, Turkey; 3 ^3^Ankara City Hospital, Department of Infectious Diseases & Clinical Microbiology, Ankara 06800, Turkey; 4 ^4^Yıldırım Beyazıt University School of Medicine, Department of Infectious Diseases & Clinical Microbiology, Ankara 06800, Turkey; 5 ^5^Yıldırım Beyazıt University School of Medicine, Department of Rheumatology, Ankara 06800, Turkey; 6 ^6^Ankara City Hospital, Department of Rheumatology, Ankara 06800, Turkey

**Keywords:** biomarkers, blood urea nitrogen/albumin ratio, co-morbidity-age-lymphocyte-LDH score, COVID-19, neutrophil/albumin ratio, predictive value of tests, SARS-CoV-2, severe acute respiratory syndrome coronavirus 2

## Abstract

**Aim:** COVID-19 is a pandemic that causes high morbidity and mortality, especially in severe patients. In this study, we aimed to search and explain the relationship between biochemical markers, which are more common, easily available and applicable to diagnose and to stage the disease. **Materials & methods:** In this study, 609 patients were evaluated retrospectively. 11 biochemical parameters were included in analysis to explain the relationship with severity of disease. **Results:** Nearly, all the parameters that have been evaluated in this study were statistically valuable as a predictive parameter for severe disease. Areas under the curve of blood urea nitrogen (BUN)/albumin ratio (BAR), CALL score and lymphocyte/C-reactive protein ratio were 0.795, 0.778 and 0.770. The BUN/BAR and neutrophil/albumin ratios provide important prognostic information for decision-making in severe patients with COVID-19. **Conclusion:** High BUN/BAR and neutrophil/albumin ratios may be a better predictor of severity COVID-19 than other routinely used parameters in admission.

## Graphical abstract

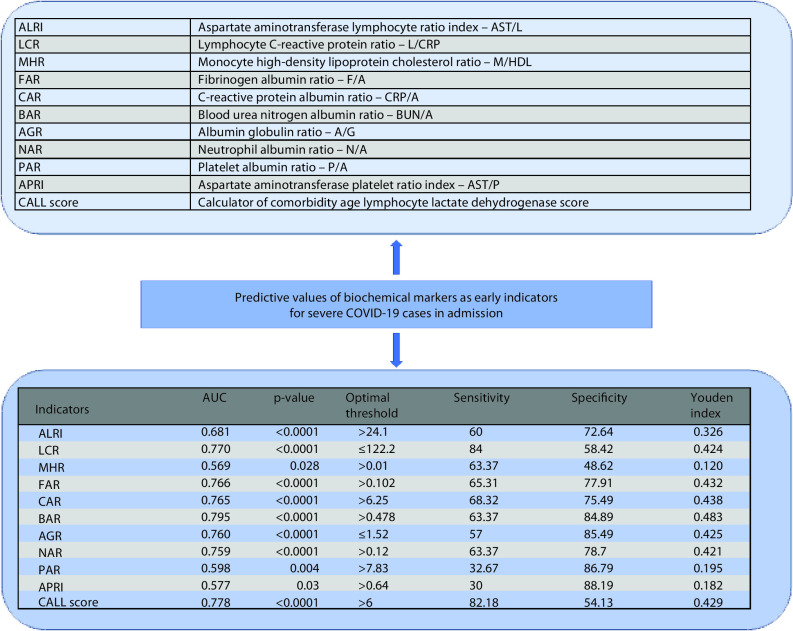

Because of the high mortality rate in severely affected patients, it is important to screen all patients with COVID-19 for hyperinflammation using laboratory parameters and/or indexes and to predict the progression of severe disease in the early period in terms of decreasing mortality.

In recent years, combined analysis of liver enzymes and platelet counts has provided important information on new biomarkers used to determine the severity of infectious and neoplastic diseases [1,2]. Moreover, thrombocytopenia and lymphopenia in severe COVID-19 patients are associated with an increased risk and even coagulopathy has poor prognosis [3,4]. Elevated aspartate aminotransferase lymphocyte ratio index (ALRI) levels prior to treatment seem to be indicators of poor prognosis in colorectal cancer [5]. The aminotransferase-platelet ratio index (APRI) has strong clinical evidence as a minimally invasive biochemical assay for detecting liver inflammation associated with various etiologies [6]. In a recent and reference study on the importance of APRI as a predictor of dengue disease severity, it was concluded that it is a more valuable predictor than independent and individual hematological − biochemical changes in distinguishing patients with severe forms of the disease [7].

Monocyte/HDL cholesterol ratio (MHR) is the new inflammatory marker currently used for cardiovascular diseases (CVDs) [8]. In addition, some studies have shown the association between MHR and CVDs such as peripheral arterial occlusive diseases, coronary artery disease (including myocardial infarction), atrial fibrillation and aortic changes [9–13].

Lymphocyte CRP ratio (LCR) is thought to be a cost-effective marker in evaluating COVID-19 complications. The LCR score is thought to indicate low immunity associated with lymphopenia and an increase in CRP [14]. It has been identified as an inflammation marker that shows the systemic inflammatory response, and lymphocyte and CRP can both be performed in nearly all laboratories [15,16].

The fibrinogen-to-albumin ratio (FAR), combined with coagulation and nutritional status, is proposed as a new marker to study prognostic value. In recent years, FAR has been shown to be positively correlated with microinflammation [17,18] and has been identified as a new clinical marker showing inflammation.

The CRP/albumin ratio (CAR) is a scoring system used to determine the degree and activity of the inflammatory disease that is thought to be more prevalent. Recently, it has been shown that CAR can be used to monitor disease activity in rheumatoid arthritis patients and as a biomarker indicating the degree of inflammatory activity in Crohn’s disease [19,20].

Recently, the blood urea nitrogen (BUN)/albumin ratio (BAR) has been found to be associated with mortality in community-acquired pneumonia (CAP) and there is a positive correlation between BAR and the severity of CAP [21–25].

Some studies have found that the albumin/globulin ratio (AGR) is associated with mortality in chronic obstructive pulmonary disease and chronic renal failure [26,27].

A multicenter study found that underlying co-morbidity, advanced age, high LDH and low lymphocyte values were independent and high-risk factors for COVID-19 progression, and it reported that the co-morbidity-age-lymphocyte-LDH (CALL) score, a new scoring system, could be a predictive value for COVID-19 progression with optimal sensitivity and specificity [28].

Platelet-albumin ratio (PAR) may represent the relationship between acute inflammation and cumulative damage in Crohn’s disease. Neutrophil-albumin ratio (NAR) has been identified as a predictor of pathologically complete response to neoadjuvant chemoradiation in rectal cancer patients [29]. NAR has been used as part of a prognostic scoring system, particularly in pancreatic cancers. As in many other parameters and ratios, PAR and NAR increase in inflammation [30].

## Objectives

Hematological and biochemical parameters are important to support the diagnosis of COVID-19. Our aim in this study was to evaluate the relationship of biochemical parameters with the confirmed group and the suspected group. We aimed to evaluate whether these biomarkers predict the severity of the disease with the first-look values in admission in COVID-19 patients. It is urgent to evaluate the capability of these features to accurately differentiate cases of COVID-19 from severe to nonsevere. Therefore, we designed the study aiming to evaluate the ability of routine laboratory tests for distinguishing COVID-19 from severe to nonsevere and help clinicians to effectively, quickly and calmly deal with COVID-19.

## Methods

In this study, 609 patients older than 18 years old who were hospitalized in the Internal Diseases and Infectious Diseases wards of a hospital in Ankara, Turkey, due to COVID-19 were evaluated retrospectively. Patients with complete data were included in the study. Patients younger than 18 years old, patients with active malignancy and pregnant women were excluded from the study. Ethical approval of the study was obtained from the Ethics Committee of a local hospital (19/08/2020, number: E1-20-998). In addition, informed consent was obtained from patients during their application to the hospital. The age, gender, co-morbidity and medications of the patients, as well as the D-dimer, fibrinogen, complete blood count, biochemical parameters, CRP, sedimentation and chest computed tomography findings at the admission were recorded. Demographic, clinical, laboratory, imaging examination, treatment and outcome data were collected using a standardized case-report form. All data were checked by two physicians (E Kaptan and B Karabuga) and then a third researcher (E Gemcioglu) determined any differences in interpretation between the two primary reviewers.

Co-morbidity means having at least one of the following: hypertension for at least 6 months, diabetes, CVD, liver disease, asthma, chronic lung disease and malignancy.

Eleven biochemical markers were used to understand the relationship with the severity of the COVID-19 patients (Table 1). CALL score is a scoring system including co-morbidity, age, lymphocyte count and lactate dehydrogenase level as parameters. Points are given as shown in Table 2 [28].

**Table 1.** Abbreviations of biochemical markers.

ALRI	AST lymphocyte ratio index – AST/L
LCR	Lymphocyte CRP ratio – L/CRP
MHR	Monocyte HDL cholesterol ratio – M/HDL
FAR	Fibrinogen albumin ratio – F/A
CAR	CRP albumin ratio – CRP/A
BAR	Blood urea nitrogen albumin ratio – BUN/A
AGR	Albumin globulin ratio – A/G
NAR	Neutrophil albumin ratio – N/A
PAR	Platelet albumin ratio – P/A
APRI	AST platelet ratio index – AST/P
CALL score	Co-morbidity-age-lymphocyte-LDH score

**Table 2.** Calculator of co-morbidity-age-lymphocyte-lactate dehydrogenase score.

	Points
**Co-morbidity** Without With	1 4
**Age (years)** ≤60 >60	1 3
**Lymphocyte** >1 × 10^9^/l ≤1 × 10^9^/l	1 3
**LDH (U/l)** ≤250 250–500 >500	1 2 3

All the patients were tested and excluded by serological tests for influenza A virus, influenza B virus, respiratory syncytial virus and parainfluenza virus. Reverse transcriptase-PCR assays were performed from the nasal and/or pharyngeal swab specimens those collected from all patients. COVID-19 diagnosis has been confirmed by the positivity of real-time PCR (RT-PCR) results.

In this study, we classified the patients into two groups according to the diagnosis and into two groups according to the stage of the disease.

In our tertiary medical facility, the patients got diagnosis either with positive PCR for COVID-19 or fulfilling four of any five of the clinical criteria including having fever, respiratory symptoms, history, compatible chest imaging findings and decreased lymphocyte count. The suspected group we mentioned are those who have the typical clinical symptoms, the typical imaging findings for COVID-19 pneumonia with negative PCR test and who have also been excluded from other infectious diagnosis or malignancy. All the patients in this study, whether they had a positive PCR or not, had COVID-19 infection. In our experience, some patients with likely COVID-19 infection may have negative results at initial RT-PCR testing.

**Figure 1.**
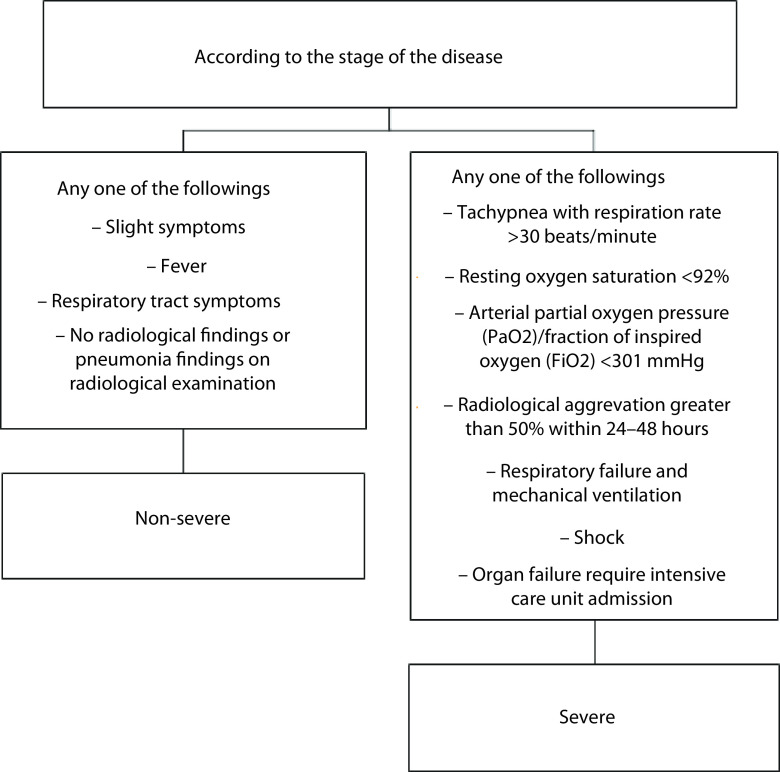
Flowchart of COVID-19 disease stage as nonsevere and severe.

According to the stage of the disease, we classified the patients into two groups as nonsevere and severe patients (Figure 1) [31,32].

To determine the severe patients in admission to the hospital for a respiratory illness, we used the slightly modified and adopted interim guidance of the WHO. The outcome of follow-up was the occurrence of severe illness, and the end of follow-up was 1 June 2020. Hospitalization, treatment, management and discharge of the patients were decided according to the guideline of the Turkish Health Ministry [33].

## Results

Of the 609 patients included in our study, 301 (49.4%) were confirmed COVID-19 patients, while 308 (50.6%) were COVID-19-suspected patients. Of these 609 patients, 348 (57.1%) were males. The median (IQR [interquartile range]) age of all patients was 49 (26.5) years (Table 3). The median (IQR) age was 55 (27) years in the suspected patient group and 45 (24.5) years in the confirmed patient group (p < 0.0001).

**Table 3.** Evaluation of the confirmed and suspected patients according to clinical status, demographics, past history and laboratory parameters other than biochemical parameters.

Characteristics or findings	All patients n: 609	Confirmed diagnosis n: 301	Suspected disease n: 308	p-value
Male sex – n° (%)	348 (57.1)	161 (53.5)	187 (60.7)	0.072
Median age – (IQR) years	49 (26.5)	45 (24.5)	55 (27)	**<0.0001**
Cough – n° (%)	321 (52.7)	179 (59.5)	142 (46.1)	**0.001**
Fever – n° (%)	228 (37.4)	142 (47.2)	86 (27.9)	**<0.0001**
Dyspnea – n° (%)	190 (31.2)	70 (23.3)	120 (39)	**<0.0001**
Headache – n° (%)	58 (9.5)	34 (11.3)	24 (7.8)	0.141
Nausea – n° (%)	41 (6.7)	23 (7.6)	18 (5.8)	0.470
Myalgia – n° (%)	132 (21.7)	83 (27.6)	49 (15.9)	**<0.0001**
Diarrhea – n° (%)	34 (5.6)	16 (5.3)	18 (5.8)	0.914
Back pain – n° (%)	3 (0.5)	2 (0.7)	1 (0.3)	0.620
Anosmia – n° (%)	35 (5.7)	24 (8)	11 (3.6)	**0.031**
Ageusia – n° (%)	28 (4.6)	20 (6.6)	8 (2.6)	**0.028**
Abdominal pain – n° (%)	11 (1.8)	2 (0.7)	9 (2.9)	0.074
Arthralgia – n° (%)	19 (3.1)	15 (5)	4 (1.3)	**0.017**
Smoking (acute and quit-smoker) – n° (%)	60 (9.9)	16 (5.3)	44 (14.3)	**0.001**
Any co-morbidity – n° (%)	264 (43.3)	99 (32.9)	165 (53.6)	**<0.0001**
Hypertension – n° (%)	169 (27.8)	63 (20.9)	106 (34.4)	**<0.0001**
Diabetes – n° (%)	107 (17.6)	39 (13)	68 (22.1)	**0.003**
Asthma – n° (%)	39 (6.4)	16 (5.3)	23 (7.5)	0.358
Obesity – n° (%)	4 (0.7)	4 (1.3)	0 (0)	0.059
CHD – n° (%)	94 (15.4)	24 (8)	70 (22.7)	**<0.0001**
Renal disease – n° (%)	34 (5.6)	5 (1.7)	29 (9.4)	**<0.0001**
ICU – n° (%)	101 (16.6)	40 (13.3)	61 (19.8)	**0.031**
Deceased – n° (%)	20 (3.3)	12 (4)	8 (2.6)	0.463
WBC (10^9^/l)	6.26 (3.41)	5.14 (2.43)	7.26 (3.8)	**<0.0001**
Lymphocyte count (10^9^/l)	1.40 (0.97)	1.24 (0.71)	1.54 (1.17)	**<0.0001**
Monocyte count (10^9^/l)	0.40 (0.23)	0.35 (0.21)	0.43 (0.28)	**<0.0001**
Hemoglobin (g/dl)	13.50 (2.60)	13.70 (2.40)	13.40 (2.70)	0.075
Platelet (10^9^/l)	231 (107)	213 (99)	246 (118.75)	**<0.0001**
Fibrinogen concentration (g/l)	3.50 (1.67)	3.3 (1.48)	3.78 (1.94)	**<0.0001**
D-dimer (mg/l)	0.5 (0.81)	0.43 (0.53)	0.6 (1.16)	**<0.0001**
CRP (g/l)	0.01 (0.04)	0.01 (0.02)	0.01 (0.04)	**0.004**
ESR (mm/h)	23 (35.5)	21.5 (29.5)	24 (40)	0.308

Note: Bold values represent that statistical significance was accepted as p < 0.05.

Statistical evaluations have been made between suspected and confirmed COVID-19 patients. All laboratory parameters have been calculated as median (IQR).

CHD: Coronary heart disease; ESR: Erythrocyte sedimentation rate; ICU: Intensive care unit; IQR: Interquartile range; WBC: White blood cell count.

Cough, fever, myalgia, anosmia, ageusia and arthralgia were statistically significantly more frequent in the confirmed patients group than in the suspected patients group (p < 0.05, Table 3). Dyspnea was significantly more frequent in the suspected group than in the confirmed group (39 vs 23.3%; p < 0.0001). There was no statistically significant difference between the suspected and confirmed group in terms of headache, nausea, diarrhea, back pain and abdominal pain.

Smoking and having any co-morbidity were statistically more common in the suspected group than in the confirmed group (14.3 vs 5.3%; p = 0.001 for smoking and 53.6 vs 32.9%; p < 0.0001 for having any co-morbidity). The need for intensive care unit (ICU) was higher in the suspected group (19.8%) than the confirmed patient group (13.3%; p = 0.031). There was no statistically significant difference in mortality between the confirmed group (4%) and the suspected group (2.6%; p = 0.463).

Considering the indexes, AGR, APRI and ALRI were significantly higher in the confirmed group than in the suspected group (p < 0.005) (Table 4). NAR, PAR, FAR, BAR, CAR, MHR and CALL points were significantly higher in the suspected group than in the confirmed group (Table 4).

**Table 4.** Evaluation of the biochemical parameters between confirmed and suspected patients at the time of admission.

Parameters	All patients n: 609	Confirmed diagnosis n: 301	Suspected disease n: 308	p-value
Creatinine (mg/dl)	0.81 (0.30)	0.80 (0.27)	0.83 (0.36)	**0.043**
BUN	14.49 (7.94)	14.02 (6.07)	15.42 (10.28)	**<0.0001**
Uric acid (mg/dl)	5 (2.3)	4.7 (2.05)	5.2 (2.4)	**<0.0001**
AST (U/l)	23 (17)	23 (15.5)	23 (18)	0.748
ALT (U/l)	27 (23)	27 (21.5)	26.5 (24.75)	0.942
LDH (U/l)	225 (100)	222 (89.75)	229.5 (123.25)	0.064
Albumin (g/l)	44 (6)	44 (6)	42 (7.75)	**<0.0001**
Globulin	24 (5)	24 (5)	24 (5)	0.331
HDL (mg/dl)	36 (16)	35 (17)	38 (16)	**0.041**
Ferritin concentration (μg/l)	121 (212.5)	115.25 (226.25)	123 (199)	0.510
ALRI	16.66 (19.67)	19.11 (19.11)	13.53 (19.32)	**<0.0001**
LCR	130.83 (559.27)	172.36 (524.39)	100 (566.81)	0.113
MHR	0.011 (0.008)	0.010 (0.008)	0.012 (0.008)	**0.003**
FAR	0.08 (0.05)	0.07 (0.04)	0.09 (0.05)	**<0.0001**
CAR^†^	2.45 (8.41)	1.70 (5.7)	3.44 (13.15)	**<0.0001**
BAR	0.33 (0.22)	0.31 (0.16)	0.36 (0.3)	**<0.0001**
AGR	1.78 (0.43)	1.82 (0.43)	1.74 (0.44)	**0.004**
NAR	0.09 (0.06)	0.07 (0.05)	0.11 (0.09)	**<0.0001**
PAR	5.32 (2.63)	4.81 (2.35)	5.88 (2.76)	**<0.0001**
APRI	0.30 (0.29)	0.32 (0.29)	0.27 (0.29)	**0.005**
CALL score	7 (5)	6 (4)	7 (4)	**<0.0001**

† CRP/albumin ratio is measured as mg/dl in unit.

Note: Bold values represent that statistical significance was accepted as p < 0.05.

Statistical evaluation has been made between confirmed and suspected patients. All laboratory parameters have been calculated as median (IQR).

AGR: Albumin/globulin ratio; ALRI: AST/lymphocyte ratio index; APRI: Aspartate aminotransferase/platelet ratio index; BAR: Blood urea nitrogen/albumin ratio; BUN: Blood urea nitrogen; CALL score: Co-morbidity-age-lymphocyte-LDH score; CAR: CRP/albumin ratio; FAR: Fibrinogen/albumin ratio; IQR: Interquartile range; LCR: Lymphocyte/CRP ratio; MHR: Monocyte/HDL ratio; NAR: Neutrophil/albumin ratio; PAR: Platelet/albumin ratio.

Detailed comparison of all three groups (all patients, confirmed patients and suspected patients) with biochemical parameters and indexes in terms of severity versus nonseverity are shown in Table 5. Severe disease patients in all three groups were older than nonsevere disease patients (p < 0.0001). Patients in the severe disease group had a higher frequency of having any co-morbidity than those in the nonsevere group (p < 0.0001).

**Table 5.** Evaluation of the biochemical parameters among confirmed, suspected and all patients according to the severity of the disease at the time of admission.

	All patients			Suspected disease			Confirmed diagnosis		
	Nonsevere n: 508	Severe n: 101	p-value	Nonsevere n: 247	Severe n: 61	p-value	Nonsevere n: 261	Severe n: 40	p-value
Male sex	291 (57.3)	57 (56.4)	0.875	152 (61.5)	35 (57.4)	0.653	139 (53.3)	22 (55)	0.972
Age, years	46 (24.75)	68 (18.5)	**<0.0001**	51 (23)	68 (19.5)	**<0.0001**	42 (21.5)	67.5 (13.75)	**<0.0001**
Any co-morbidity	195 (38.4)	69 (68.3)	0.875	120 (48.6)	45 (73.8)	<0.0001	75 (28.7)	24 (60)	<0.0001
Creatinine (mg/dl)	0.8 (0.26)	0.91 (0.77)	**0.001**	0.8 (0.26)	1.05 (1.08)	**<0.0001**	0.8 (0.27)	0.82 (0.34)	0.704
BUN	14.02 (7.01)	20.09 (20.8)	**<0.0001**	14.49 (7.94)	21.96 (30.37)	**<0.0001**	13.08 (5.75)	19.39 (12.73)	**<0.0001**
Uric acid (mg/dl)	4.90 (2.2)	5.5 (3)	**0.003**	5.10 (2.40)	5.85 (2.75)	**0.017**	4.70 (2)	5.2 (3.3)	0.234
AST (U/l)	22 (16)	28 (27)	**0.001**	23 (18)	24 (23.25)	0.637	22 (14)	32 (42.5)	**<0.0001**
ALT (U/l)	27 (22.75)	24 (28.5)	0.321	29 (24)	20 (21)	**0.009**	26 (20)	29 (34.5)	0.074
LDH (U/l)	221 (85)	285 (210.75)	**<0.0001**	225 (94.5)	280 (216)	**0.001**	217 (80.5)	291 (193)	**<0.0001**
Albumin (g/l)	44 (6)	38 (8.50)	**<0.0001**	43 (6)	36 (8.5)	**<0.0001**	45 (5)	38 (8.75)	**<0.0001**
Globulin	24 (5)	25 (6)	**0.043**	24 (5)	25 (5.5)	0.062	24 (4.25)	24 (6)	0.436
HDL (mg/dl)	37 (15)	32 (17)	**0.002**	38 (15)	35 (18.35)	0.071	36 (17)	30.5 (14.1)	**0.005**
Ferritin (μg/l)	112 (193)	188 (489.5)	**<0.0001**	118 (194)	152.5 (455)	**0.012**	99.5 (196.25)	272 (488.5)	**<0.0001**
ALRI	15.22 (16.04)	27.78 (38.05)	**<0.0001**	12.63 (15.37)	24.86 (30.91)	**0.001**	17.71 (15.84)	40.91 (74.38)	**<0.0001**
LCR	186.93 (636.51)	21.24 (82.29)	**<0.0001**	156.43 (834.09)	25.10 (57.38)	**<0.0001**	208.13 (566.88)	16.30 (150.72)	**<0.0001**
MHR	0.011 (0.008)	0.012 (0.009)	**0.029**	0.012 (0.008)	0.013 (0.010)	0.243	0.010 (0.007)	0.012 (0.009)	0.093
FAR	0.08 (0.04)	0.12 (0.08)	**<0.0001**	0.09 (0.05)	0.12 (0.1)	**<0.0001**	0.07 (0.03)	0.11 (0.08)	**<0.0001**
CAR^†^	1.69 (5.79)	14 (25.49)	**<0.0001**	2.40 (8.44)	14.63 (20.61)	**<0.0001**	1.32 (4.21)	12.82 (33.68)	**<0.0001**
BAR	0.31 (0.17)	0.6 (0.71)	**<0.0001**	0.33 (0.2)	0.63 (0.95)	**<0.0001**	0.3 (0.13)	0.51 (0.43)	**<0.0001**
AGR	1.83 (0.41)	1.46 (0.53)	**<0.0001**	1.80 (0.41)	1.46 (0.51)	**<0.0001**	1.87 (0.39)	1.46 (0.59)	**<0.0001**
NAR	0.08 (0.06)	0.15 (0.15)	**<0.0001**	0.11 (0.07)	0.17 (0.14)	**<0.0001**	0.07 (0.05)	0.13 (0.10)	**<0.0001**
PAR	5.2 (2.46)	5.91 (4.5)	**0.002**	5.77 (2.47)	6.52 (4.26)	0.06	4.74 (2.12)	5.45 (4.02)	**0.034**
APRI	0.28 (0.26)	0.37 (0.5)	**0.014**	0.26 (0.25)	0.3 (0.42)	0.536	0.31 (0.27)	0.43 (0.57)	**0.001**
CALL score	6 (4)	9 (4)	**<0.0001**	7 (5)	9 (4)	**<0.0001**	5 (3)	10 (4)	**<0.0001**

† CRP/albumin ratio is measured as mg/dl in unit.

Note: Bold values represent that statistical significance was accepted as p < 0.05.

Statistical evaluation has been made between confirmed and suspected patients. All laboratory parameters have been calculated as median (IQR).

AGR: Albumin/globulin ratio; ALRI: AST/lymphocyte ratio index; APRI: AST/platelet ratio index; BAR: Blood urea nitrogen/albumin ratio; BUN: Blood urea nitrogen; CALL score: Co-morbidity-age-lymphocyte-LDH score; CAR: CRP/albumin ratio; FAR: Fibrinogen/albumin ratio; IQR: Interquartile range; LCR: Lymphocyte/CRP ratio; MHR: Monocyte/HDL ratio; NAR: Neutrophil/albumin ratio; PAR: Platelet/albumin ratio.

According to the severe versus nonsevere grouping, a statistically significant difference was found in the ‘all patients’ group for all 11 indexes (Table 5).

A multivariate logistic regression model for severe disease consisted of the variables age, male gender and 11 indexes in Table 6. In the multivariate logistic regression analyses, NAR in the highest tertile (odds ratio [OR]: 228.775; 95% CI: 6.850–7640.711; p = 0.002) was determined as an independent predictor of severe disease in COVID-19. On multivariate analyses, the serum BAR, AGR, APRI and age were found to be the independent predictors of severe disease in COVID-19.

**Table 6.** Evaluation of the biochemical parameters of all patients according to the severity of disease with multivariate logistic regression analyses at the time of admission.

Parameters	All patients		
	OR	95% CI	p-value
Male sex	1.246	0.696–2.231	0.459
Age, years	1.051	1.031–1.072	**<0.0001**
Any co-morbidity	0.736	0.387–1.400	0.350
ALRI	0.996	0.992–1.000	0.078
LCR	1.000	0.999–1.000	0.206
MHR	0.000	0.000–7.131	0.077
FAR	0.997	0.530–1.875	0.992
CAR^†^	1.010	0.994–1.027	0.216
BAR	2.185	1.151–4.148	**0.017**
AGR	0.486	0.291–0.812	**0.006**
NAR	228.775	6.850–7640.711	**0.002**
PAR	1.089	0.995–1.191	0.065
APRI	1.418	1.059–1.900	**0.019**
CALL score	1.158	0.938–1.429	0.172

† CRP/albumin ratio is measured as mg/dl in unit.

Note: Bold values represent that statistical significance was accepted as p < 0.05.

All laboratory parameters have been calculated as median (IQR).

AGR: Albumin/globulin ratio; ALRI: AST/lymphocyte ratio index; APRI: AST/platelet ratio index; BAR: Blood urea nitrogen/albumin ratio; CALL score: Co-morbidity-age-lymphocyte-LDH score; CAR: CRP/albumin ratio; FAR: Fibrinogen/albumin ratio; IQR: Interquartile range; LCR: Lymphocyte/CRP ratio; MHR: Monocyte/HDL ratio; NAR: Neutrophil/albumin ratio; OR: Odds ratio; PAR: Platelet/albumin ratio.

The values of 11 combinations of inflammatory markers and other biochemical indexes in all patients with severe disease of COVID-19 were calculated and the predicted values of these parameters were compared in the receiver-operating characteristic (ROC) analysis. In Table 7, areas under the curve (AUC) of BAR, CALL points and LCR were 0.795, 0.778 and 0.770, respectively. The optimal cutoff values were >0.478, >7 and ≤122.2 for BAR, CALL points and LCR, respectively. All biochemical parameters could be used as potential diagnostic biomarkers for subsequent analysis because their AUC was higher than 0.50.

**Table 7.** The areas under the curve and optimal thresholds of each independent risk or protection factors for biochemical parameters of all patients according to the severity of disease.

Indicators	AUC	p-value	Optimal threshold	Sensitivity	Specificity	Youden index
ALRI	0.681	**<0.0001**	>24.1	60	72.64	0.326
LCR	0.770	**<0.0001**	≤122.2	84	58.42	0.424
MHR	0.569	**0.028**	>0.01	63.37	48.62	0.120
FAR	0.766	**<0.0001**	>0.102	65.31	77.91	0.432
CAR^†^	0.765	**<0.0001**	>6.25	68.32	75.49	0.438
BAR	0.795	**<0.0001**	>0.478	63.37	84.89	0.483
AGR	0.760	**<0.0001**	≤1.52	57	85.49	0.425
NAR	0.759	**<0.0001**	>0.12	63.37	78.7	0.421
PAR	0.598	**0.004**	>7.83	32.67	86.79	0.195
APRI	0.577	**0.03**	>0.64	30	88.19	0.182
CALL score	0.778	**<0.0001**	>6	82.18	54.13	0.429

† CRP/albumin ratio is measured as mg/dl in unit.

Note: Boldface values represents that statistical significance was accepted as p < 0.05.

All laboratory parameters have been calculated as median (IQR).

AGR: Albumin/globulin ratio; ALRI: AST/lymphocyte ratio index; APRI: AST/platelet ratio index; AUC: Areas under the curve; BAR: Blood urea nitrogen/albumin ratio; CALL score: Co-morbidity-age-lymphocyte-LDH score; CAR: CRP/albumin ratio; FAR: Fibrinogen/albumin ratio; IQR: Interquartile range; LCR: Lymphocyte/CRP ratio; MHR: Monocyte/HDL ratio; NAR: Neutrophil/albumin ratio; PAR: Platelet/albumin ratio.

## Discussion

The ability of MHR to predict the anti-inflammatory effect is based on the proinflammatory–prooxidative effects of monocytes and the anti-inflammatory effect of HDL, especially on the endovascular system [34]. Increased MHR has been shown to correlate with high levels of high-sensitivity CRP [8,35]. Canpolat *et al.* [11] reported that MHR is associated with diffuse atherosclerosis, inflammation and microvascular dysfunction, and reduces coronary flow in CVDs. Microvascular dysfunction may be observed in patients with severe COVID-19 [36]. Considering all patients of our study, high MHR level was found to be an indicator of severe disease. However, when the patient groups were considered, no significant difference was found between the severe and nonsevere groups in terms of MHR. In addition, we did not find MHR measured at the first admission as an independent risk factor for predicting severe disease.

ALRI has been found to be associated with prognosis, particularly in liver disease and hepatocellular carcinoma (HCC), and its higher rate in postoperatory HCC patients indicates the poor prognosis [37]. In our study, the ALRI value in severe patients was found to be significantly higher than in the nonsevere group. There is no study in the literature evaluating the relationship between ALRI and COVID-19. However, the elevation of AST in mitochondrial liver damage secondary to inflammation and the fact that lymphopenia is an indicator of poor prognosis in COVID-19 patients suggested that the increase of this rate may be important in predicting severe patients, but ALRI on admission was not accepted as an independent risk factor for severe cases of COVID-19 in our study.

As a noninvasive biochemical assay, APRI was found to be important in determining the degree of liver damage and fibrosis in chronic liver patients, chronic hepatitis B and C patients in many studies [38–44]. Its high level in HCC, nonalcoholic liver disease, metastatic colorectal cancer patients is associated with prognosis [45–49]. In a study by Guan *et al.*, high AST levels were observed in severe COVID-19 cases due to liver dysfunction [50]. Lippi *et al.* showed that a low platelet count is associated with an increased risk matched to signs of thrombocytopenia in patients with severe COVID-19 [51]. When all COVID-19 patients were evaluated in our study, APRI was found to be higher in the severe patients group. APRI on admission was accepted as an independent risk factor for severe cases of COVID-19 in our study.

It indicates the state of immune-inflammation in patients with LCR malignancies [14,52,53]. It is thought that preoperative LCR dysregulation may predict the perioperative surgical risk and postoperative oncologic outcomes in oncologic surgery patients [14]. LCR is a marker of inflammation, reflecting the systemic inflammatory response [52,53]. A published meta-analysis reported that the LCR value decreased significantly in severe COVID-19 patients [54]. Similar to this meta-analysis, the LCR level was significantly lower in the severe group compared with the nonsevere group in our study. The LCR value was 122.2 and its sensitivity in predicting severe disease was 84%, while its specificity was 58% (CI: 0.735–0.803; p < 0.0001).

Platelets are an acute-phase reactant induced by major inflammatory cytokines, including IL-1, IL-6, INF-γ and TNF, and can be used as a crude indicator of inflammatory activity [55,56]. Some other studies have shown that severe COVID-19 patients have significantly decreased albumin levels [55,57], but the change in albumin does not parallel the severity of hepatocellular damage in COVID-19 [55]. This is thought to be due to intense systemic inflammation and increased capillary permeability. In a study conducted in China, it was reported that a hypoalbumin level of <35 g/l increased mortality six-times independently in COVID-19 patients [58]. Stidham *et al.* reported in their study that PAR might be an indicator of activity in Crohn’s patients and might predict the need for surgery. Similarly, in our study, which supports all these studies, the PAR value was found to be significantly higher in severe COVID-19 patients. But in the ROC analysis, although the AUC was significant, it was lower than the other parameters (AUC [CI]: 0.598 [0.558–0.638]; p = 0.004).

**Figure 2.**
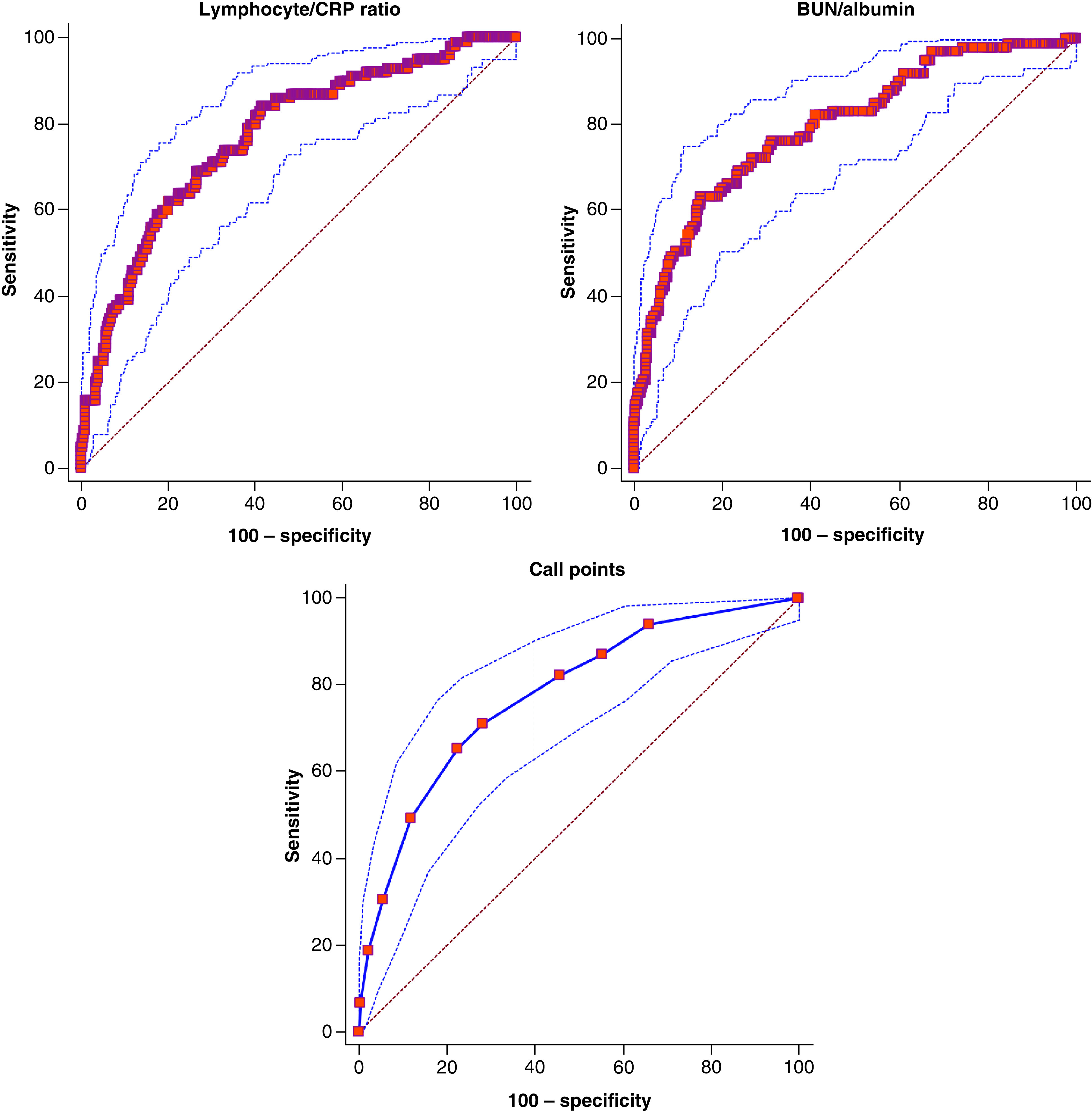
Receiver-operating characteristic curve of lymphocyte/CRP ratio, blood urea nitrogen/albumin ratio and co-morbidity-age-lymphocyte-LDH score.

Subtypes of white blood cell count (WBC) alone are good predictors for the inflammation. A meta-analysis showed that neutrophil count was positively related to the severity of COVID-19. NAR was found to be the independent predictor of survival in pancreatic cancer. Similarly, in our study, we found that NAR was significantly higher in COVID-19 patients than in nonsevere groups. In ROC analysis, the AUC of NAR was found to be CI: 0.759 (0.723–0.793), while the cutoff value was more than 0.12, which is the best for predicting severe disease (p < 0.0001). When we evaluated with all biomarkers in logistic regression, we found that NAR had the highest OR in predicting severe disease: OR (CI) 228.775 (6.850–7640.711); p = 0.002 (Figure 2).

Fibrinogen is not only an essential component of the coagulation cascade but also an acute-phase reactant that demonstrates inflammation [59]. Fibrinogen plays a key role in the inflammation process, leading to the synthesis of proinflammatory cytokines involved in the inflammatory environment and inflammation in the peripheral blood [60]. FAR is widely used as an effective marker of inflammation, and there are different studies showing that it has prognostic value in severe infections, cardiovascular events [61], cancers [62,63], myocardial infarctions [64] and contrast-associated nephropathies [63,65]. In many studies on SARS-CoV and MERS-CoV, it has been stated that hypercoagulation and fibrinolysis increase the risk of microthrombi and aggravate organ failure [66]. It is thought that elevated FAR levels may be associated with cytokine storm induced by virus invasion [67]. Bi *et al.* reported in their study that high FAR levels of severe COVID-19 patients were closely associated with disease progression [68]. In the same study, it was reported that the FAR level in severe COVID-19 patients was higher than in nonsevere patients and that it gradually returned to the normal level as the patient recovered [68]. Similarly, in our study, FAR was found to be significantly higher in the severe patient group compared with the nonsevere patient group. When FAR value was more than 0.102, the AUC was: CI 0.766 (0.730–0.800; p < 0.0001). In a recent study, FAR and platelet count were identified as independent risk factors predicting severe disease in COVID-19 patients. It has been reported that patients with FAR <0.0883 and platelet count >135 * 109/l are at a low risk of developing severe disease.

It has been reported that levels of CRP above 130 mg/l may be associated with increased mortality [3]. Albumin levels are significantly lower in severe COVID-19, but the change in albumin levels does not parallel the severity of hepatocellular damage. This suggests that hypoalbuminemia may have another cause other than hepatocellular damage. One of these causes is the intense systemic inflammation reported in severe COVID-19. Hypoalbuminemia is common in many inflammatory diseases because increased capillary permeability causes albumin leakage into the interstitial space [69]. CAR is an index that has been found to be high in many infections, malignancies and inflammations and shows the severity of the disease [70–73]. CAR, alone, indicates the status of inflammation better than CRP or albumin. Similarly, in our study, CAR was found to be significantly higher in the severe patient group compared with the nonsevere patient group. Although the AUC value of CAR was 0.765, it could not be detected as an independent risk factor in predicting severe disease.

According to some studies, patients who died of CAP had higher BUN levels compared with survivors [74], while albumin levels of these patients were found to be low. Ugajin *et al.* found that the increase in the BAR in patients admitted to hospital with CAP positively correlated with the severity of the disease and increased mortality [75]. In our study, the BUN level in severe patients was found to be significantly higher than in the nonsevere group. In our study, the BAR was also found to be significantly higher in patients with severe disease. The BAR performed better in detecting severe disease than other parameters. The BAR was also found to be an independent predictor of severe disease: OR (CI): 2.185 (1.151–4.148); p = 0.017.

Globulin is a reflection of all nonalbumin proteins, including other prothrombotic proteins and inflammatory proteins. Changes in serum globulins are thought to be indicative of inflammation and/or host immune response [76]. A study in the literature found that both CVD-related and independent mortality was high in chronic kidney disease patients with low AGR [26]. It has been stated in different studies that AGR can be used in the prognostic evaluation of tumors such as HCC, ureteral carcinoma, ovarian and gastric cancer [77,78]. AGR can also be used as an index to assess mortality from myocardial infarction [79]. In our study, the decrease in the AGR value in the severe patient group was found to be statistically significant. We identified AGR as an independent risk factor predicting severe disease. High level of AGR at admission is important in terms of nonsevere disease progression.

Studies on COVID-19 have reported that age, co-morbidities, lymphopenia, serum ferritin, D-dimer levels, cardiac troponin I, lactate dehydrogenase and IL-6 subsets are associated with poor prognosis and increased mortality [28,80–83]. CALL score is a scoring system calculated according to co-morbidity, age, lymphocyte count and LDH level. Low risk (CALL score 4–6 points) group had less than 10% probability, intermediate risk (CALL score 7–9 points) group had 10–40% probability, high risk (CALL score 10–13 points) group had more than 50% probability for progression, respectively [28] With the CALL score, it is aimed to predict the prognosis and clinical progression of the disease and help clinicians in this regard. It has been reported that the CALL scoring system, which has four clinical parameters, is simpler than the 12-parameter MuLBSTA score proposed by Guo *et al.* [84]. In our study, the CALL score was significantly higher in the severe disease group. When the cutoff value was more than 7, which is the best for the CALL score, the sensitivity was 71.65% and the specificity was 71.65%. In the article in which the CALL score is defined, using a cutoff value of 6 points, the positive and negative predictive values were 50.7 and 98.5%, respectively [28]. In our study, when we accepted the cutoff for the CALL score as >6, we found that the sensitivity increased to 82.18%, while the specificity decreased to 54.13%.

The confirmation diagnosis of COVID-19 is based on PCR tests to detect SARS-CoV-2 but diagnosis of the COVID-19 patient with negative PCR assays depends primarily on clinical, imaging and epidemiological features. Similarly, in our study, significant results were obtained when comparing the confirmed and suspected groups of COVID-19 patients. Dyspnea was significantly higher in the suspected patient group than in the confirmed patient group. This result is not surprising considering that one of the inclusion criteria of the suspected patient group is radiological examination. In addition, the patients in the suspected group were older and smoking and co-morbidities were most frequent in this group. Another interesting result in our study was that the need for intensive care was higher in the suspected group. This may be due to the fact that smoking and co-morbidities were more frequent in this group, and the patients in this group were older.

Our research had some limitations. First, it was performed in a single center and was a retrospective study. For validation, results of this study require multiple neutral prospective studies. Second, only the combination of 11 inflammatory markers was discussed to predict the severity of COVID-19 patients, and future studies may consider incorporating additional biomarkers in the same manner. The third limitation for this study was that all the markers and measurements were evaluated just one-time on admission, therefore changes in those parameters could not be evaluated. Our last limitation is that the introduction and discussion sections of our article are a bit long since we have examined 11 indexes together.

The advantage of our study compared with other current studies is that almost all the parameters (biomarkers) in the literature have been studied together in our study. It can be said that our study is different since both the number of parameters and the number of patients are higher than in other studies. In addition, the fact that biochemical markers were evaluated in both confirmed and suspected groups is another difference for our study.

The values of 11 combinations of inflammatory markers in patients with COVID-19 were calculated, and the predicted values of these ratios were compared in the ROC analysis. The AUC values for the BAR were the highest among the 11 combinations of inflammatory markers. CALL points and lymphocyte/CRP ratio are the other inflammatory markers that have high AUC values following the BAR.

Considering both the ROC analysis and the multivariate analysis together, NAR and BAR are one step ahead in terms of predicting disease severity compared with other biochemical markers. Measurement of cytokine level is difficult to be routinely applied in state hospitals for COVID-19, as they are not easily accessible and are expensive. The fact that NAR and BAR can be used routinely is another feature that highlights these parameters.

## Conclusion

The BAR and NAR provide important prognostic information for decision-making in patients with severe COVID-19. High BAR and NAR may be a better predictor of COVID-19 than other routinely used parameters in admission. All patients with COVID-19 should be screened for severity using BAR and NAR to reduce mortality. Our findings indicate that the parameter those enhanced from routine biochemical examination, which is a simple laboratory test, can help to identify and classify COVID-19 patients into nonsevere to severe groups.

By using the index thresholds given in Table 7, especially during pandemic outbreaks, we believe that clinicians can triage cases with severe disease more easily and faster.

**Summary points**

- COVID-19 – severe acute respiratory syndrome coronavirus 2 (SARS-CoV-2) − is a pandemic infectious disease that causes morbidity and mortality. The prognosis of the disease may range from complete well being to severe acute respiratory distress syndrome or death.
- In this study, we included the patients at first presentation who were diagnosed as COVID-19 and we tried to find the easily applicable index to determine the severity of the diseases at the presentation.
- Nearly, all the parameters that have been evaluated in this study were statistically valuable as a predictive parameter for severe disease.
- In the multivariate logistic regression analyses, NAR in the highest tertile (odds ratio: 228.775; 95% CI: 6.850–7640.711; p = 0.002) was determined as an independent predictor of severe disease in COVID-19. On multivariate analyses, the serum BAR, AGR, APRI and age were found to be the independent predictors of severe disease in COVID-19.
- Areas under the curve of BAR, CALL points and LCR were 0.795, 0.778 and 0.770, respectively. The optimal cut-off values were >0.478, >7 and ≤122.2 for BAR, CALL points and LCR, respectively. All biochemical parameters could be used as potential triage biomarkers for subsequent analysis because their areas under the curve was higher than 0.50.
- Our findings indicate that the parameter those enhanced from routine biochemical examination, which is a simple laboratory test, can help to identify and classify COVID-19 patients into nonsevere to severe groups.
